# Nectar Feeding by a Honey Bee’s Hairy Tongue: Morphology, Dynamics, and Energy-Saving Strategies

**DOI:** 10.3390/insects12090762

**Published:** 2021-08-24

**Authors:** Hao Wang, Zhigang Wu, Jieliang Zhao, Jianing Wu

**Affiliations:** 1 School of Aeronautics and Astronautics, Sun Yat-Sen University, Guangzhou 510006, China; wangh659@mail2.sysu.edu.cn (H.W.); wuzhigang@mail.sysu.edu.cn (Z.W.); 2 School of Mechanical Engineering, Beijing Institute of Technology, Beijing 100081, China; 6120190098@bit.edu.cn

**Keywords:** honey bee, mouth parts anatomy, nectar feeding behaviour, dynamics, energy-saving strategies

## Abstract

**Simple Summary:**

This paper reviews the interdisciplinary research on nectar feeding behaviour of honey bees ranging from morphology, dynamics, and energy-saving strategies, which collects a range of knowledge of feeding physiology of honey bees and may inspire the design paradigms of next-generation multifunctional microfluidic transporters.

**Abstract:**

Most flower-visiting insects have evolved highly specialized morphological structures to facilitate nectar feeding. As a typical pollinator, the honey bee has specialized mouth parts comprised of a pair of galeae, a pair of labial palpi, and a glossa, to feed on the nectar by the feeding modes of lapping or sucking. To extensively elucidate the mechanism of a bee’s feeding, we should combine the investigations from glossa morphology, feeding behaviour, and mathematical models. This paper reviews the interdisciplinary research on nectar feeding behaviour of honey bees ranging from morphology, dynamics, and energy-saving strategies, which may not only reveal the mechanism of nectar feeding by honey bees but inspire engineered facilities for microfluidic transport.

## 1. Introduction

The majority of flower-visiting insects, including bees, wasps [1,2], flies [3], butterflies [4], moths [5], and some beetles [6,7], obtain nutrition from floral nectar and pollen from flowering plants [8]. The honey bee (*Apis mellifera ligustica*) is a typical pollinator in the world [9]. The specialized proboscis is of great importance for a honey bee to load nectar rapidly and efficiently. The mouth parts of a honey bee are comprised of a pair of galeae, a pair of labial palpi, and a glossa [6]. The honey bee performs two feeding modes, namely lapping and suction [10]. While lapping, the honey bee drives its segmented tongue (glossa) coated in dense hairs back and forth to load nectar. When the honey bee dips nectar, the glossa protracts with the glossal hairs adhered to the glossa body. Then the glossa reaches to the maximum extension with the glossal hairs deployed. Next the brush-like glossa is filled with nectar and retracts to the mouth parts to load nectar. While sucking, the glossa extends out of the proboscis tube, directly sucking with the glossa keeping still [10].

Honey bees can feed on a range of viscous fluids at high efficiencies [8]. This behaviour is challenging because of the physical property of nectar, suggesting the nectar viscosity increases steeply with respect to the concentration, through which the glossa should have to resist high viscous drag [11,12,13,14]. In addition, if the glossa dips faster, the energetic intake rate will augment; however, the energy consumption caused by viscous drag will increase, so honey bees should have to meet the contradictive demands of both high energetic intake rate and low energetic loss while feeding on nectar. Investigations of the honey bee’s feeding behaviour and related mechanical principles may reflect the health status of bees and adaptations to environmental constraints. More extensively, a healthier bee may consume less energy while feeding on nectar, who might be able to optimize the nectar harvest due to the mechanism of drag reduction. In addition, combined biological and mathematical analysis on feeding behaviour of bees may even elucidate the co-evolution between flowering plants and nectarivorous insects. In this review, we will introduce some interdisciplinary problems associated with honey bee’s feeding behaviour. We will start with the anatomy of the mouth parts, followed by feeding modes, mechanism of hair erection, and energy saving strategies and conclude with potential engineering applications. The rest of the paper is structured as follows. Section 2 introduces the anatomical structure of the honey bee’s mouth parts. Section 3 illustrates feeding fashion of a honey bee glossa from the perspectives of glossa kinematics and drag reduction mechanism, and Section 4 introduces the energy saving strategy by the glossa’s dynamic surface. Functional compensation by regulating dipping frequency is shown in Section 5. Section 6 includes conclusions.

## 2. Honey Bee Mouth Parts Morphology

**Figure 1:**
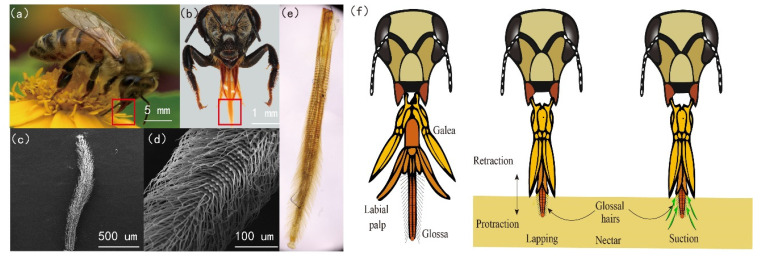
The honey bee’s mouth parts. (**a**) A honey bee feeding on nectar on a flower. (**b**) The head and mouth parts of a honey bee. The mouth parts, highlighted in a red box, are comprised of a pair of galeae, a pair of labial palpi, and a glossa (*Apis mellifera* L.). (**c**) Scanning electron microscopic images of a bee glossa. (**d**) The glossa with bushy hairs. (**e**) The glossa observed under a microscope. (**f**) Lapping and sucking modes of a honey bee. The galeae and labial palpi form the probocid tube, then the glossa makes reciprocating movements through the tube to lap nectar [16].

For the western bee (*Apis mellifera* L.), the mouth parts are comprised of a pair of galeae, a pair of labial palpi, and a hairy glossa, namely, the glossa in length of 2 mm (Figure 1a–c) [6,12]. Bushy glossal hairs in length of 100 μm, with diameter of 1~3 μm, are attached to the surface of the kidney-shaped sheath, which appear annulated on the glossa (Figure 1c–d). A thin membrane is attached to the edges of the sheath, which is also next to the corresponding sides of the muscular rod inside the glossa [15]. Notably the glossa has ~120 segments and is structured in a compliant manner. In the centre of glossa, a humour-filled cavity is formed by the sheath, muscular rod, and thin membranes. The honey bees are described to have two feeding modes, namely lapping and suction [10]. For the lapping mode, the glossa moves forward and backward with glossal hairs erecting rhythmically to load the nectar (Figure 1f). For the suction mode, the glossa stays still through the proboscis tube, and the nectar is sucked up by the cibarial pump, generating flows across the glossa surface [10]. 

## 3. Feeding Behaviour of a Honey Bee Glossa

### 3.1. Section-Wise Wettability of the Glossa

**Figure 2:**
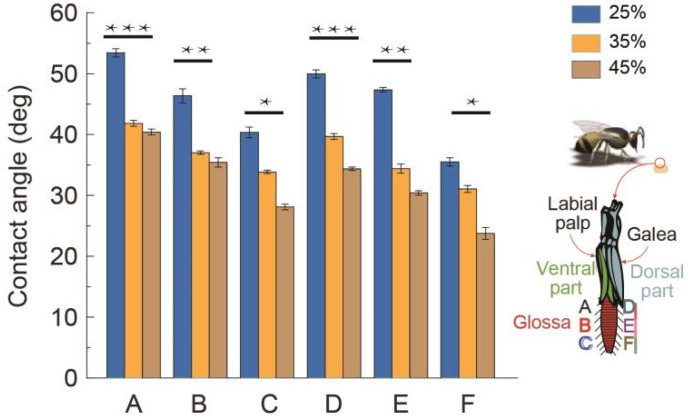
Section-wise wettability of a honey bee’s glossa. Six regions of the glossa immersed in nectar, marked with A~F, and A~C, and D~F, represent the ventral part and dorsal part respectively. Contact angles of different regions on the glossa surface of 25%, 35%, and 45% sucrose solution. The glossa surface is more hydrophilic to the higher-concentration nectar, which is elucidated from the decreasing of contact angles with the increased nectar concentration are shown in the histogram [21]. Asterisks indicates the comparison groups.

Wettability is the ability of surface to be wetted by liquid, which is determined by the balance of surface energy in the interface between air, liquid, and solid materials [17]. A honey bee propels its glossa to lap the viscous nectar, so the wettability of a honey bee glossa may be related to the nectar trapping capability [18], and the contact angle (wetting angle) is a measure of the wettability of a solid by a liquid. Generally, if the water contact angle is smaller than 90°, the solid surface is considered hydrophilic and inversely if the contact angle is bigger than 90°, the solid surface is considered hydrophobic. As a result, it is necessary to test the wettability of a honey bee glossa before we examine its feeding capability [19]. For bee flowers, the average nectar concentration in nature is 36% [20], so 25%, 35%, and 45% sucrose solutions were prepared for lab tests. Under a microscope, the contact angles measured in different glossal regions are shown in Figure 2. The results indicate that the contact angles turn smaller when using the sucrose solution with higher concentration, which insinuates that the surface exhibits stronger hydrophilicity to the thicker nectar. More extensively, the ranking of section-wise hydrophilicity suggests that the dorsal side is much easier to be wetted than the ventral side (Figure 2). We calculated the *p*-value as 0.03 between the two data sets of contact angles on the ventral surface and the dorsal surface, respectively, which suggests a significant difference between these two data sets, denoting that the dorsal surface is much easier to be wetted than the ventral surface for the reason of that the ranking of hydrophilicity, namely, D > A, E > B, and F > C. In order to better understand the comparison groups, we indicate them by stars shown in Figure 2. Moreover, the glossa tip is more hydrophilic than the middle region of the glossa, and the proximal part is the hardest to be wetted by the nectar. The section-wise wettability of the glossa may be caused by the chemical and geometrical differences on these hairy segments; the glossa surface is more hydrophilic to the higher-concentration nectar, which might be beneficial for nectar trapping, especially for the dynamic glossa surfaces. The combined chemical and geometrical differences on these hairy segments may contribute to a high flexibility in adaptation to varying environments, for instance, a broad range of liquid viscosities found in floral sources. In the next subsection, we will introduce the feeding pattern of honey bees via high-speed filming under the conditions of foraging on nectar with varying viscosities. 

### 3.2. Facultative Feeding Modes in Honey Bees

**Figure 3:**
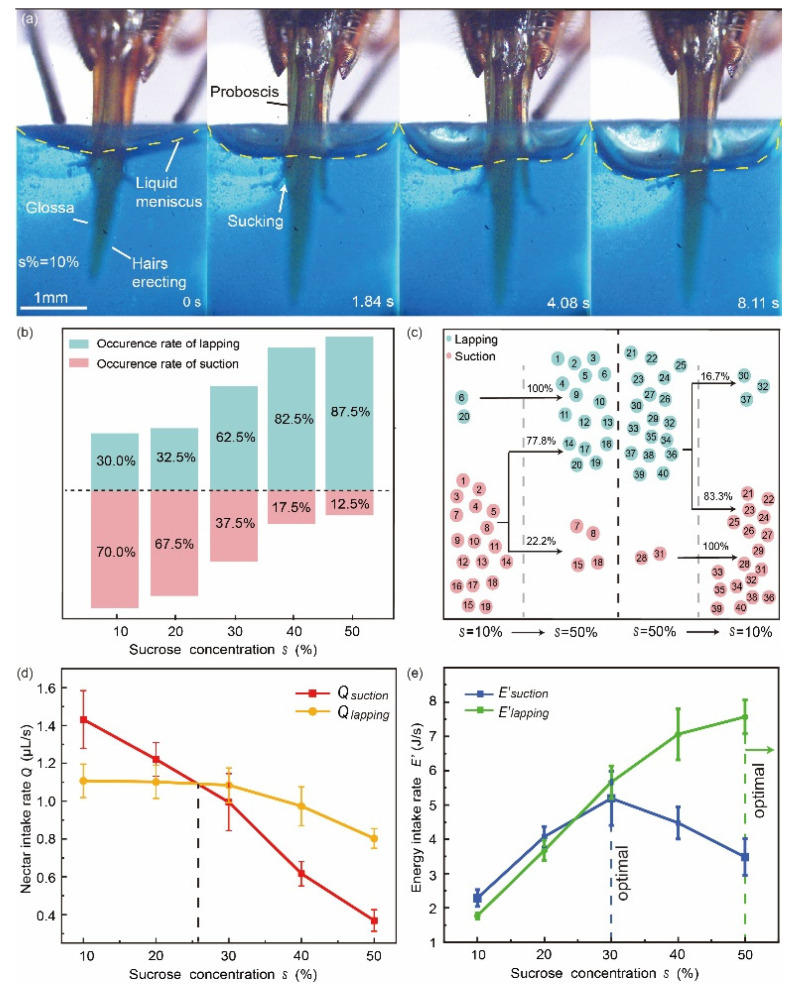
Switchable feeding pattern in a honey bee. (**a**) High-speed images of a honey bee sucking the artificial nectar. (**b**) Occurrence rates of the two feeding modes in honey bees, when feeding on sucrose solutions with various concentrations [10]. (**c**) Occurrence rates of switching between feeding modes when offered extreme nectar concentrations, and the dotted lines represent binary feeding mechanisms in various nectar concentrations. Each encircled number represents a different individual. (**d**) Nectar intake rates of suction and lapping under different concentrations nectar, dashed line denotes the equivalent point of feeding efficiency and the corresponding sugar concentration, the dotted line denotes an equal nectar intake rate of the feeding modes under a specific nectar concentration. (**e**) Energy intake rates of suction and lapping under different nectar concentrations. Blue dashed line depicts the optimal concentration for suction mode, and green dashed line denotes that the optimal concentration for lapping mode is around 50% or above.

The feeding pattern of a honey bee was previously defined as “lapping”, which refers to reciprocating movements of its glossa entraining nectar by the glossal hairs. However, bees would also directly suck nectar with glossa keeping protracting and staying still (Figure 3a). Wei et al. [10] demonstrate that bees have facultative feeding behaviour. Bees prefer to suck the nectar with low sugar concentration and they tend to lap the nectar with more sugar content (Figure 3b). Further lab tests showed that honey bees can switch between these two feeding patterns to choose a more efficient ingesting mode (Figure 3c). The capillary-based lapping mechanism that allows honey bees to achieve high energy intake rates when feeding on highly concentrated nectar [22], while sucking directly with glossa protracting and staying still facilitates feeding on less viscous nectar (Figure 3d,e), besides the energy intake rate *E*′ was calculated by *E*′ = *ρscQ*/100, where *ρ* denotes the density of the nectar, *s* the sugar concentration, *c* the energy content per unit mass of sugar, and *Q* the nectar intake rate. Experiments validated that the key stimulus of choosing the ingesting technique is the viscosity of the nectar, rather than sugar content, according to the result that most bees feed on nectar with 10% sugar concentration, but with viscosity equivalent to 50% concentration (by adding Tylose) exhibited lapping pattern. This facultative drinking mode that is behaviourally adjusted to fluid viscosity has potentially enhanced the adaptability of honey bees to a wider range of nectar resources [23,24,25].

### 3.3. Kinematics of the Glossa and Glossal Hairs

For the lapping mode, the glossa extends out of the proboscid tube structured by the labial palpi and galeae, with glossal hairs attaching on the glossa body. Then the glossa moves back into the proboscis tube and glossal hairs flatten to offload the nectar. Here is a kinematic asymmetry, in which glossa protracts faster in a spear-like shape and retracts more slowly in a brush-like configuration. This asymmetry functions as a strategy to save energy, especially reducing the energetic consumption induced by viscous fluidic drag [26]. By observing kinematics of the glossa and glossal hairs by high-speed filming, Zhao [16] further found a specific asynchronization between glossa movements and glossal hair erection. A physical model is proposed to describe the feeding process considering the trade-off between nectar-intake volume and energy consumption. This asynchronization may be caused by the material properties of the elastic rod and the compliance of the segmented structures, especially the zig-zag shaped intersegmental membranes of the glossa [12], which is validated to be effective in maximizing the nectar-intake amount by theoretically figuring out the optimal moment when the glossal hairs begin to erect. This asynchronization suggests that the honey bee glossa can perform a scheduled coordination between glossa movements and hair erection, which could serve as valuable models for developing miniature pumps that are both extendable and have dynamic surfaces. 

**Figure 4:**
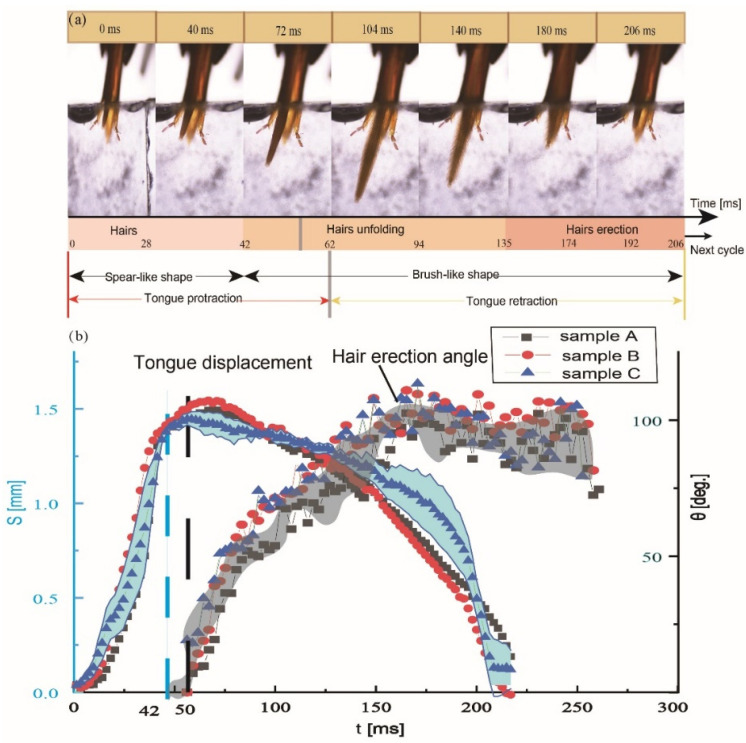
Morphological changes in glossal surfaces during dipping nectar and surface configurations through stretching the honey bees’ glossae. (**a**) Dipping pattern of a honey bee tongue. (**b**) Asynchronization between tongue displacement and average hair erection angle. Both the in vivo and postmortem observations reveal that shortening and lengthening of the glossal segments is highly coordinated with the erection of glossal hairs, which aids in developing deformable gaps between rows of glossal hairs during nectar trapping [16].

To uncover the anatomical mechanism of the coordination of glossa extension and hair erection, Zhu [27] compared hair erection and segment elongation and discovered a high consistency of their kinematics during the drinking process (Figure 4). In a dipping cycle, when the average erection angle of glossal hairs increases from 20 deg. to 38 deg., the average length of one glossal segment increases from 22.9 ± 1.6 μm to 24.7 ± 2.2 μm. The concordance equation was applied for evaluating correlation between these variables. The concordance measure is equal to 0.99 in the in vivo observation experiments, which shows that the average elongation of a glossal segment is closely correlated to the average erection angle of hairs.

**Figure 5:**
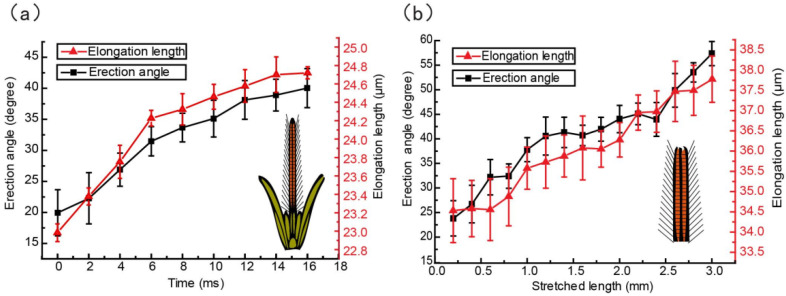
Coordinated movements of hair erection and segment elongation of the glossa. (**a**) Natural behaviour of nectar drinking. (**b**) Highly-coordinated movements of the glossal segments and hair erection through stretching the glossa under a microscope [27].

To further demonstrate the relationship between glossa protraction and hair erection, Zhu [27] stretched the glossae of honey bees and observed them under a microscope. Glossal hairs of a dead honey bee’s glossa erect only when the glossa is stretched by an external force, suggesting that the elongation of the glossal segments is coordinated with the hair erection (Figure 5). The average erection angle of hairs varies from 23 deg. to 57 deg., and the average length of one glossal segment increases from 34.7 ± 2.8 μm to 37.7 ± 3.1 μm accordingly. The concordance value is calculated as 0.96, which is close to the observations on living animals (0.99). This highly-coordinated motion indicates that the glossal hairs are hinged in the intersegmental membranes, which could deploy and fold synchronously. 

### 3.4. Coordinated Movements of the Abdomen While Dipping Nectar

**Figure 6:**
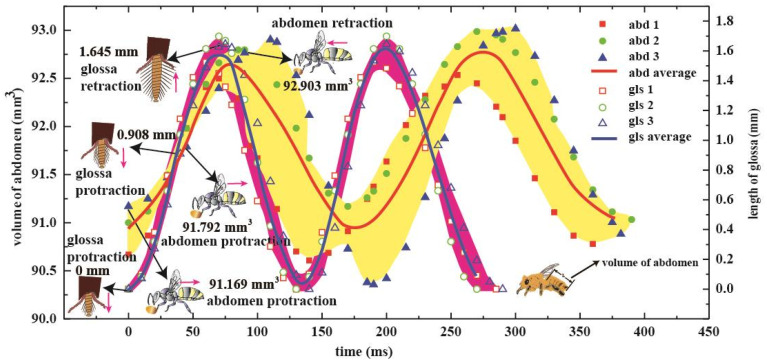
Dependence of abdominal and glossal movements (three samples are shown here). Both the glossa and abdomen protracted and retracted periodically, the pink arrow denotes the direction of protraction and retraction of the glossa and abdomen, thereby showing an approximate sinusoidal principle. The left vertical axis shows the volume of abdomen when lapping nectar against time, and the right vertical axis shows the real-time length of the glossa [15].

As a lapper, honey bee uses a mop-like glossa to trap nectar from flowering plants. By filming the feeding honey bees, a significant increase in abdominal pumping frequency was observed when honey bees drink the sucrose solution [16]. Zhao [16] combined high-speed filming, X-ray phase contrast imaging, and mathematical models to investigate the effect of abdominal pumping in liquid feeding of honey bee. A honey bee performs abdominal pumping during feeding, which is in concordance with reciprocating movements of the glossa (Figure 6). The modelling framework demonstrates that the abdominal pumping powers the honey bee’s feeding efficiency and saves foraging time. The combined experimental and theoretical investigations extend the knowledge about the function of abdominal movements, which is considered only for adjusting flight attitude or crawling through honeycombs [28]. This behaviour is functionally analogous to power suction feeding in some fish that uses most power of axial swimming muscles not only by the cranial muscles [29]. The multifunctional use of muscular actuations fulfils the switchable requirements of these animals and makes the organs structurally compacted and efficient. 

## 4. Energy Saving Strategy

For the viscous dipping mode, a honey bee should have to meet the combined requirement of both high energetic intake rate and low energetic dissipation caused by viscous drag. A honey bee may have to make millions of reciprocating movements during its whole life, so an energy-saving mechanism may be required to reduce the energy consumption and lower the possibility of wear caused by the viscous drag. This section includes some interdisciplinary work that covers morphology, high-speed imaging, and lubrication models, to uncover the energy saving strategy while feeding on nectar [30]. 

### 4.1. Modelling for Energy Saving

**Figure 7:**
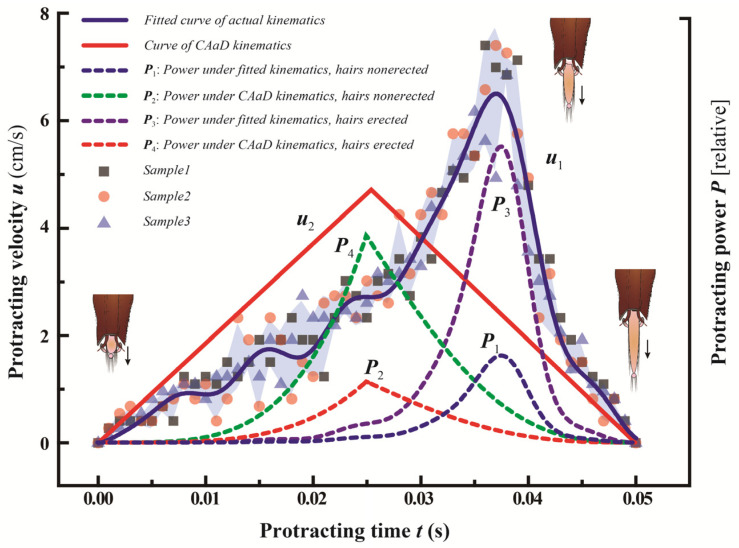
Energy saving by specific glossa kinematics. The scattered points show three independent protraction velocities measured from the high-speed video and the light blue area indicates the error band of the velocities. The bold blue curve represents the Fourier kinematics *u*_1_, which fit the scatter plot well. The bold red curve shows the constant-acceleration-and-deceleration (CAaD) kinematics *u*_2_. The dotted blue line *P*_1_ and the dotted red line *P*_2_ indicate the protraction power under the fitted kinematics and constant-acceleration-and-deceleration kinematics, respectively [26].

Figure 7 shows the actual glossa kinematics and hypothetical cases with various kinematic apportionment. A 7-order Fourier function that fits the actual glossa velocity in a dipping cycle (*R*^2^ = 0.9853) is shown as

(1) $$u\left(t\right)=K_{1}f\left(t\right)=K_{1}\left(a_{0}+\sum_{i=1}^{7}\left(a_{i}\cos\left(\omega t\right)+b_{i}\sin\left(\omega t\right)\right)\right)$$

where *f*(*t*) is the fitting equation; *a*_0_, ω, *a*_i_ and *b*_i_ (1 ≤ *i* ≤ 7) are the parameters calculated by Matlab to obtain the best fit for the scatter plot; and *K*_1_ (137 μm/cm) is a coefficient that links the sizes in high-speed photographs into those for an actual honey bee. It can be demonstrated that the honey bee can reduce its energy expenditure using the derived protraction kinematics. The power required for resisting viscous drag can be estimated as *P*_v_~*μLu*^2^, where *μ* is the nectar viscosity and *L* is the glossa length during protraction, and the power to drive glossa can be estimated as *P*_t_~*mu*’*u*~*ρ*_t_*a*^2^*u*^3^, where *a* and *ρ*_t_ are the radius of the glossa and the density of the glossa, respectively. Since the ratio *P*_t_/*P*_v_ ≪ 1, the effect of *P*_t_ can be neglected [26]. The viscous drag can be written as $F_{v}\propto \mu \cdot \left(2\pi a\right)\cdot x\left(t\right)\cdot u\left(t\right){=K}_{2}\mu a\cdot x(t)\cdot u(t)$, where *K*_2_ is a proportionality coefficient. Combining Equation (1) and the formula *x*(*t*) = $\int_{0}^{t}f(t)dt$, the power needed to overcome viscous drag reads.

(2) $$P_{v}\left(t\right){=F}_{v}\cdot u\left(t\right){=\mu aK}_{2}K_{1}^{3}f{\left(t\right)}^{2}\overset{t}{\underset{0}{\int }}f\left(t\right)dt$$

One can evaluate the benefits of keeping the glossal hairs still during tongue protraction from Equation (1). If the honey bee erects the hairs, the outer diameter of the glossa radius will augment from *a* to (*a* + *h*cos*θ*), which will lead to a significant increase in *P*_v_(*t*). Scanning electron microscope imaging indicates *a* ≈ 50 μm and *h* ≈ 170 μm, and since *θ* ≈ 45°, we then arrive at (*a* + *h*cos*θ*)/*a* = 3.4, which means that hair erection increases the resistance by more than three times. Therefore, the honey bee is equipped with a specific glossal hair erection pattern for energy saving, where the flatten hairs can reduce viscous drag during protraction, whereas the hairs erect to trap more nectar in a single cycle during retraction. When a glossa makes reciprocating movements through viscous fluid, the viscous drag will exert on the hairy glossa surface. The nectar is a specific solution, the physical property of which is analogous to the sucrose solution, and its viscosity rises steeply with respect to concentration. From the perspective of Fluid Mechanics, the viscous drag dissipates energy so we should have to consider the energy dissipation linked to viscous drag. Some previous tests were made to validate the fact that more viscous nectar causes higher rate of wear, which indicates the viscous drag can accelerate the structural deterioration on the seemingly fragile glossal hairs. 

### 4.2. Effects of Galea Ridges on Drag Reduction

Biological surfaces with unique microstructures in nature may perform specific functions, such as impact absorption and drag reduction in dung beetles or sharks, respectively [31]. The honey bee, *Apis mellifera* L., dips viscous nectar at a high rate which is about 5 Hz by the glossa, which causes non-negligible fluidic drag that results in structural and functional deterioration. By postmortem examination, Li [32] found the ridges are parallel distributed on the inner wall of the galeae and validated its effects on drag reduction. Li then compared the structural discrepancy between workers and drones and proposed some implications about the caste-related behaviour [32]. 

**Figure 8:**
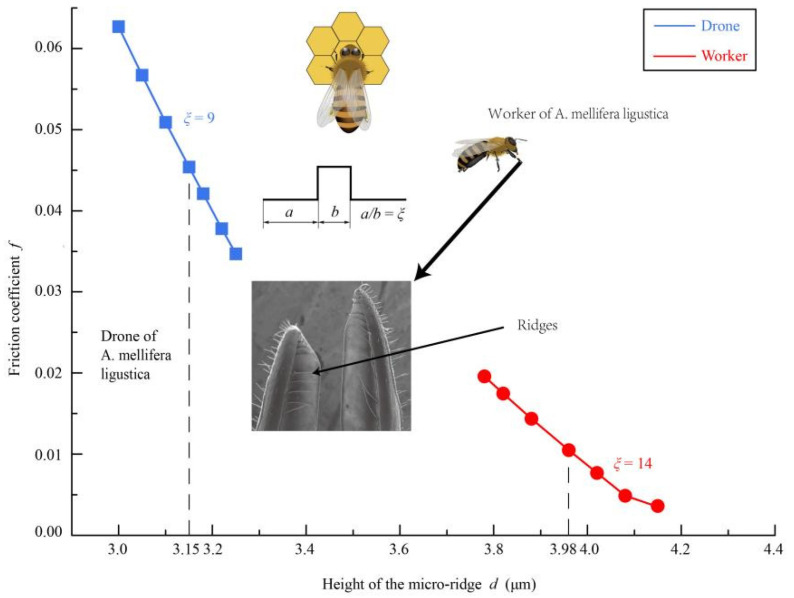
The friction coefficient against the heights of the micro-ridges on the inner wall of the galeae of workers and drones. The blue square and red dot represent the friction coefficient against the heights of the microridges on the inner wall of the galeae of workers and drones, respectively. Here *a* and *b* denote the dimensions of the microridges of different castes of honey bees, in which *a* is the length of the galea and *b* is the ridges, and ξ denotes the average dimensions of the workers and drones. The dotted lines illustrate the measured mean height of the microridges on the galeae of the workers was 3.98 μm, whereas that of the drones was 3.15 μm [32].

Scanning electron microscopy (SEM) images indicate that the honey bee galea has internally transverse ridges uniformly distributed (Figure 8). Theoretical analysis show that the ridges on the galeae of honey bee’s mouth parts of workers can reduce the friction coefficient by 86%. Li [32] then examined the dimensional diversities of the uniformly-distributed micro-ridges on inner walls of galeae among workers and drones of *Apis mellifera* L. The hydrodynamic model was used to calculate the friction coefficient in the mouth parts, further testing whether the sexually-dimensional variations of the micro-ridges could influence the effect on drag reduction. Theoretical estimations of the friction coefficient with respect to the dipping frequency show that the inner micro-ridges can significantly reduce friction during the feeding process of a honey bee. Li then compared effects of drag reduction regulated by the sexually-selected micro-ridges and demonstrated that the hydrodynamic coefficients of workers and drones are 0.011 ± 0.007 and 0.045 ± 0.010 respectively, which indicates that workers exhibit better capability of drag reduction in their mouth parts than that of drones. This discrepancy may have some more indications in caste-related work of honey bees. The main physiological requirement of drones is to find an airborne queen to mate and accordingly, so drones exhibit strong adaptations to forceful flying, and drones possess elaborate mating organs and powerful sense organs, such as big eyes and long antennae with many receptors for visual and olfactory orientations toward airborne queens [33]. Thus, although drones have bigger bodies, their mandibles are shorter, and their stomachs for honey storage are slimmer than those of workers [34]. Compared to drones, workers should have to fulfil a variety of tasks [35]. Workers tidy the hive, care the brood, nourish the larvae, drones, and the queen, and work for nest homeostasis [36,37]. Given these various duties, workers are equipped with well-developed hypopharyngeal and possess longer mouth parts than drones. Notably, adult drones are nourished by worker-prepared food, and their feeding ability is weaker than that of workers [34]. This experimental and theoretical combined research elucidated that the sexually-selected micro-ridges, developed inside workers and drones of honey bees’ mouth parts, are structurally adapted to meet the demands of caste-related laborers of honey bees.

## 5. Functional Compensation by Regulating Dipping Frequency

**Figure 9:**
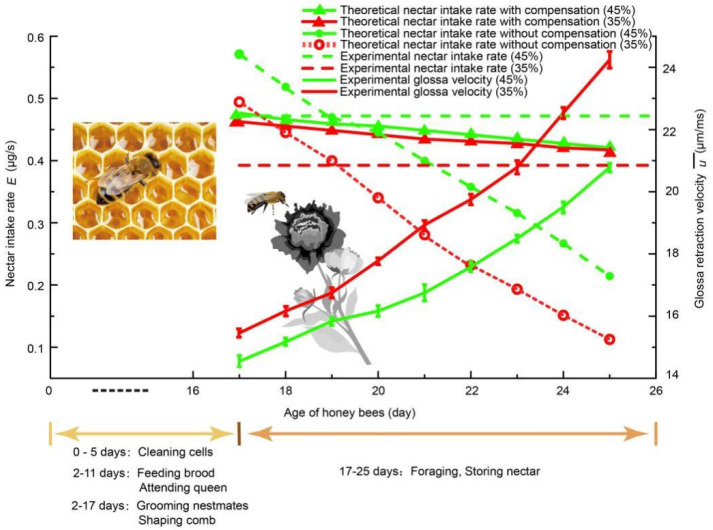
The honey bee augments dipping frequency to compensate for glossa hair deterioration. The relationship between theoretical nectar mass intake rate $\dot{M}$ and setae wear agrees with the experimental data captured from lab tests for dipping both the 35% and 45% sucrose solutions [39].

Because of the highly-intensive viscous drag exerting on the glossa during nectar feeding, the glossal setae tend to wear out in the high-viscosity nectar. However, bees at varying day ages can maintain the nectar intake rate at 0.39 ± 0.03 μg·s^−1^ (35% nectar). Shi found that the average glossal setae length decreases with respect to age from 17 to 25 days, and it degrades even faster when fed with higher-viscosity nectar. Lab tests indicated that the older honey bees with short setae dip nectar more quickly. Moreover, a correlation between dipping frequency *f* and the average glossal setae length *h*, is found as *h* = −15.435*f* + 212.04. Based on the glossa anatomy, a fluid transport model is proposed to calculate the nectar intake rate. Theoretical analysis showed that a honey bee with shorter setae can compensate the nectar intake rate by increasing the dipping frequency. Considering the wear of the setae and dipping compensation, Shi arrived at the results that the total energy intake rate is about 106 times the power required to overcome viscous drag; the energy dissipation caused by viscous drag is negligible [26]. Therefore, the effect of augmentation of viscous drag caused by the increase of the dipping frequency on the energy intake rate of bees is almost negligible. Natural selection tends to feed quickly and efficiently, as honey bees are threatened by predators and economic necessities [38]. Therefore, honey bees must meet the contradictive demands of keeping the visit time short and the optimal nectar mass intake rate. Although the natural wear of glossal setae will affect the nectar intake rate, by adjusting the dipping frequency, both requirements can be satisfied, which is in accordance with the results from lab tests of wearing bee tongues in the 35% and 45% sucrose solutions, respectively (Figure 9). 

## 6. Conclusions

Investigations of feeding techniques by a honey bee’s glossa are interdisciplinary work that covers morphology, behavioural dynamics, and energy-saving strategies. Future work might be extended to the following aspects. (1) The nectar property may drive the feeding property more complicated, in which, for instance, the nectar viscosity increases steeply with respect to the nectar concentration, and it is also influenced by the temperature [13]. The flowers may have an internal microclimate which is up to 4 °C higher than the external temperature, which not only provides more heat to sustain the thoracic temperature of honey bees especially in winter time but makes the nectar a bit thinner, which is much easier to be digested because of the lower nectar viscosity [13]. The combined experimental and theoretical methodology is required to uncover this. (2) The dipping behaviour may be an indicator to reflect the health state of the honey bees. Air pollution, pesticide abuse, and climate change may strongly influence the honey production rate and even survival rate of the bee colony [40,41]. The dipping frequency is closely related to the energetic intake rate, so we may use the dipping frequency as a measure to evaluate the health status of the bee colony. (3) The bee cannot only feed on nectar in different floral structures but can lick dry sugar during droughts. The functional flexibility in feeding remains unexplored. The bee glossa is comprised of segmented structure which can perform a million times of reciprocating movements. How the bee glossa meets the contradictive demands of high deformability and stiffness is still unknown. Combining various experiments and theoretical frameworks, more extensive research will be conducted, not only to reveal the behavioural characteristics of honey bees but for inspiring the next-generation facilities like micropumps and other viscous fluidic transport facilities.

## Data Availability

Data is contained within the article or supplementary material. The data presented in this study are available in “Nectar feeding by a honey bee’s hairy tongue: morphology, dynamics, and energy-saving strategies”.
